# Retraction Note: GAS5 knockdown alleviates spinal cord injury by reducing VAV1 expression via RNA binding protein CELF2

**DOI:** 10.1038/s41598-022-26451-0

**Published:** 2022-12-20

**Authors:** Dan Wang, Xiaoxiao Xu, Junwei Pan, Shixin Zhao, Yu Li, Zhen Wang, Jiahao Yang, Xi Zhang, Yisheng Wang, Ming Liu

**Affiliations:** Department of Orthopedic, The First Affiliated Hospital of Zhengzhou University, No. 1, Jianshe East road, Erqi District, Zhengzhou, 450052 China

Retraction of: *Scientific Reports* 10.1038/s41598-021-83145-9, published online 11 February 2021

The Editors have retracted this Article.

After publication of this Article concerns were raised about the veracity of the data presented here. The Authors were unable to provide all the raw data and requested retraction. The Authors also didn't respond to Editors' subsequent request to provide evidence of ethics approval. The Editors therefore no longer has confidence in the integrity of the data in this Article.

The Authors did not respond to the Editors' correspondence about the retraction notice.

